# Retraction Note: Exploring the potential effect of COVID-19 on an endangered great ape

**DOI:** 10.1038/s41598-022-05893-6

**Published:** 2022-01-25

**Authors:** Fernando Colchero, Winnie Eckardt, Tara Stoinski

**Affiliations:** 1 Department of Mathematics and Computer Science, University of Southern Denmark, Campusvej 55, 5230 Odense, Denmark; 2 Interdisciplinary Center On Population Dynamics, University of Southern Denmark, Campusvej 55, 5230 Odense, Denmark; 3 The Dian Fossey Gorilla Fund, 800 Cherokee Ave SE, Atlanta, GA 30315 USA

Retraction of: *Scientific Reports* 10.1038/s41598-021-00061-8, published online 21 October 2021

The Authors have retracted this Article.

It was brought to our attention that we had made an error in our simulation study of the potential effect of SARS-CoV-2 on a subpopulation of the Virunga mountain gorilla population. Specifically, instead of using human infection fatality rates (IFRs) as a reference for our simulations, we used case fatality rates (CFRs) from studies published early during the pandemic in Wuhan, China, and Italy, adjusted to the longevity of the mountain gorillas. Case fatality rates, particularly those early during the pandemic, have since been found to be considerably higher than the actual infection fatality rates. After repeating our analyses with this updated information and using the adjusted CFRs instead of IFRs, we found that our published results significantly overestimated the chances of extinction of the population should a COVID-19 outbreak occur. Although the revised findings are important for mountain gorilla conservation, they substantially change the conclusions of the study. In light of these important discrepancies, we deem it necessary to retract this study.

All Authors agree with this retraction and its wording.

